# Status and Influencing Factors of Social Media Addiction in Chinese Medical Care Professionals: A Cross-Sectional Survey

**DOI:** 10.3389/fpsyg.2022.888714

**Published:** 2022-04-27

**Authors:** Aijing Luo, Weitao Kong, Haiyan He, Yuanyuan Li, Wenzhao Xie

**Affiliations:** ^1^Key Laboratory of Medical Information Research, The Third Xiangya Hospital, Central South University, Changsha, China; ^2^Clinical Research Center for Cardiovascular Intelligent Healthcare, The Second Xiangya Hospital, Central South University, Changsha, China; ^3^Key Laboratory of Medical Information Research (Central South University), College of Hunan Province, Changsha, China; ^4^School of Life Sciences, Central South University, Changsha, China; ^5^Department of Geratology, Hunan Provincial People’s Hospital, The First Affiliated Hospital of Hunan Normal University, Changsha, China

**Keywords:** social media addiction, burnout, general self-efficacy, medical care professionals, cross-sectional survey

## Abstract

**Background:**

In modern society, social media addiction (SMA) has become a serious problem in many countries, including China. Almost every medical care professional has their own social media account. They are also at risk for SMA, but no SMA studies in Chinese medical care professionals have been published. This study aims to investigate the status and influencing factors of SMA among Chinese medical care professionals.

**Methods:**

A cross-sectional study was conducted among 519 physicians and nurses from two randomly selected hospitals using a questionnaire that included the Social Networking Service Addiction Scale (SNSAS), Maslach’s Burnout Inventory-General Survey (MBI-GS), the General Self-efficacy Scale (GSES), and eight demographic datasets.

**Results:**

This study’s findings showed that most of the participants’ (357,68.79%) scores reached 2.5 points (half of the highest possible score), indicating that SMA scores of Chinese medical care professionals were relatively high. Significant differences in SMA scores by age (*p* < 0.01), marital status (*p* < 0.01), professional title (p < 0.01), and working years (p < 0.01) were found. Income satisfaction (p < 0.01) and sleep quality (*p* < 0.05) were negatively correlated with SMA. The GSES score was not correlated with SMA (*p* = 0.377). Burnout significantly positively affected SMA (*p* < 0.01).

**Conclusion:**

Our study found that the SMA scores of Chinese medical care professionals were relatively high. To reduce the SMA level of the medical care population, we should first start with reducing burnout, enabling medical care professionals to achieve sufficient sleep, increasing medical staff income, and providing more opportunities for promotion.

## Introduction

After decades of technological progress, the media industry has changed how information is disseminated and the way people socialize. Social media networks on smart devices allow users to contact friends anytime and anywhere (Kuss and Griffiths, 2011) and make new friends (Ma et al., 2018). They can also exchange information, express opinions, express their emotions, share their current situation, and understand other people’s lives through social media (Mantymaki and Islam, 2016). In modern China, social media has become ubiquitous and transformative (DeLisle et al., 2016). According to Tencent data, 94% of WeChat users log in every day, 61% of users open WeChat more than 10 times a day, and 55% of users are online for more than 1 h a day (Penguin Intelligence, 2016). The figures are only from WeChat, and other social media apps are popular in China, such as QQ, Sina Weibo, and Tieba. However, social media is not harmless. Unreasonable and excessive use of social media can interfere with other aspects of daily life and can also lead to addiction.

Social media addiction (SMA) is a kind of technology addiction, manifested in excessive attention to social media, strong motivation to use social media, and a large amount of energy to use social media, so as to affect physical health, mental health, interpersonal relationship, study and work efficiency and life happiness (Andreassen and Pallesen, 2014). Social media addicts cannot control their attention to and use of social media (Andreassen and Pallesen, 2014). In recent years, the negative impact of social media has become increasingly obvious, and social media addiction (SMA) has become a serious problem (Elgan, 2015). Unrestrained use of social media can affect mental health (Chen and Lee, 2013), which may cause anxiety and depression (Demirci, 2019), an increase in the level of cyberloafing (Turan et al., 2021), and a negative impact on romantic (Tokunaga, 2014) and other interpersonal relationships (Bevan et al., 2014). In addition to mental health, physical health can also be negatively affected by the unrestrained use of social media (Xue et al., 2018). This new type of technology addiction is prevalent in dozens of countries with different cultures and values around the world (Cheng et al., 2021). In China, symptoms of SMA are found in different groups, including adolescents (Huang et al., 2021), college students (Hou et al., 2019), and information technology employees (Khan et al., 2021). In the information era, almost every medical care professional has their own social media account (Kung and Oh, 2014; Low et al., 2020), but no SMA studies have been performed in Chinese medical care professionals.

**Figure 1:**
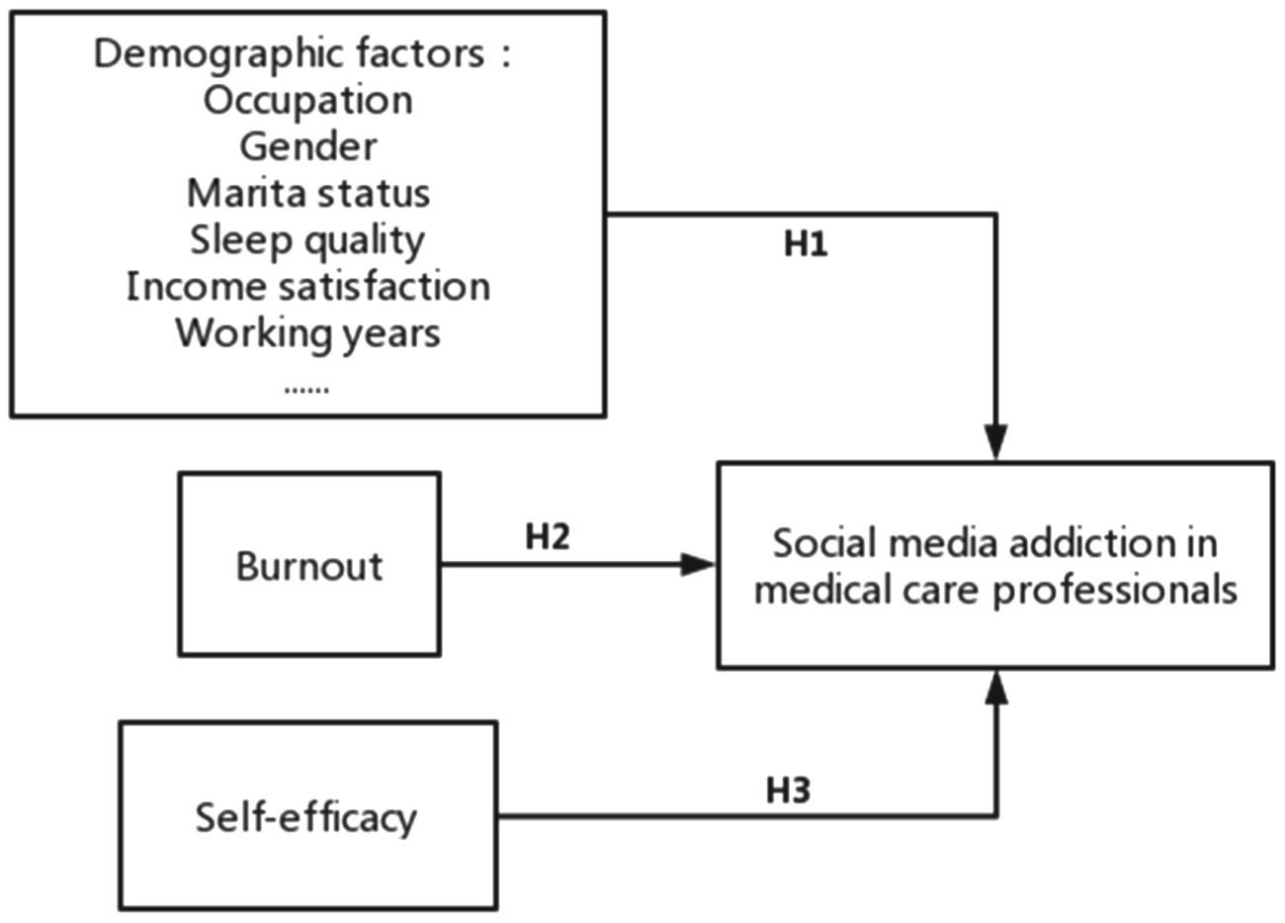
Influencing factors model of social media addiction (SMA) in medical care professionals.

After reviewing the previous literature, we found that social media addiction is a kind of technology addiction, which may be related to some demographic factors (Lenhart, 2015; Andreassen, 2015; Kawyannejad et al., 2019; Sharifinia et al., 2019; Aslan and Yasar, 2020) and may also be influenced by burnout (Chen et al., 2014; Younes et al., 2016; Zivnuska et al., 2019) and self-efficacy (Seo et al., 2015; Im, 2017). Based on these potential influencing factors, we established a model of the influence relationship of SMA (Figure 1).

### Demographic Determinants of SMA

Previous studies have found that social media addiction may be related to gender (Aslan and Yasar, 2020), age (Andreassen, 2015; Lenhart, 2015), marital status (Sharifinia et al., 2019), sleep quality (Kawyannejad et al., 2019) and other demographic factors. In addition, professional position, income satisfaction, working years and other factors that may be closely related to medical care groups are added, we propose hypothesis 1.

*H1*: The level of SMA in Chinese medical care professionals was correlated with demographic factors (Occupation, Gender, Age, Marital status, Professional position, Income satisfaction, Sleep quality, Working years).

### Burnout

Freudenberger HJ proposed the concept of burnout in 1974 (Freudenberger, 1974). Currently, the meaning of burnout is expressed as an imbalance between the effort invested in the job and the results obtained (Farber, 1983) or a response to work stress and work overload (Gil-Monte, 2002; Ortega and López, 2004; Carlin and Ruiz, 2010). Burnout is a psychosomatic syndrome characterized by emotional exhaustion, depersonalization, and a reduced sense of personal accomplishment (Maslach et al., 2001). Burnout, which is mainly caused by high work stress, is widespread among Chinese medical care professionals, and its incidence is high (Zhang et al., 2021), showing a younger age profile (Wu et al., 2007). Burnout is common among doctors (Ma et al., 2020) and is also an important issue among Chinese nurses (Lin et al., 2009), representing a problem that warrants attention (Cui et al., 2013). Burnout is more severe in the intensive care unit (Wang et al., 2021). Outside of the high-intensity work environment of urban hospitals, staff members in grassroots medical institutions in China, such as doctors in rural areas and community nurses, also experience burnout (Hu et al., 2015; Xu et al., 2020; Zhao et al., 2021). Stress is associated with technology addiction. Work stress is associated with an increased risk of internet addiction (Chen et al., 2014), and the two are significantly correlated (Younes et al., 2016). Academic pressure may cause internet addiction (Jun and Choi, 2015) and is an important risk factor for internet addiction in medical students (Radeef and Faisal, 2018). The internet is a place to escape stress (Gong et al., 2021; Monacis et al., 2021). Recent research has also linked social media addiction to factors such as job burnout and stress. For example, SMA has been associated with lower productivity at work (Zivnuska et al., 2019), as well as levels of employee distraction (Majid et al., 2020), work environment and work atmosphere (Moqbel and Kock, 2018). Monitoring employees at work can also cause SMA problems (Choi et al., 2019). Consider the potential impact of burnout on social SMA, we propose hypothesis 2.

*H2*: Burnout levels were positively correlated with SMA levels in Chinese medical care professionals.

### Self-Efficacy

Self-efficacy is the core of a social cognitive theory (Bandura, 1977). Self-efficacy refers to personal views and beliefs when faced with difficulty. In a broad sense, self-efficacy refers to a broad and stable sense of one’s ability to effectively cope with various stressful situations (Sherer et al., 1982; Schwarzer and Jerusalem, 1995). In China, some scholars have conducted investigations on the self-efficacy of nurses and found that the self-efficacy scores are relatively high for orthopedic nurses (Dai et al., 2021) and moderate for midwives (Jiang et al., 2020). The self-efficacy of Chinese nurses is related to peer support (Wang et al., 2018) and scientific research capability (Jiang et al., 2019). Self-efficacy has been shown to have a negative impact on technology addiction. A strong negative correlation has been identified between excessive internet use/internet addiction and self-efficacy (Berte et al., 2021). People with higher self-efficacy scores have stronger self-control on the internet (Iskender and Akin, 2010). In contrast, people with low self-efficacy are more likely to suffer from internet addiction (Chen H. -C. et al., 2020); the same is true of game addiction (Jeong and Kim, 2011). Self-efficacy may mediate the relationship between academic stress and interpersonal stress and smartphone addiction (Chiu, 2014), and decreased self-efficacy can lead to addiction to smartphones (Lee et al., 2018). In a recent study from South Korea, researchers found that college women’s self-efficacy was negatively correlated with their SMA level (Seo et al., 2015). Considering the influence of self-efficacy on technology addiction, we propose hypothesis 3:

*H3*: Self-efficacy level was negatively correlated with SMA level in Chinese medical care professionals.

### Gaps in the Literature

Although research on social media addiction has made great strides in the past decade or so, several critical gaps remain in the literature. In recent years, most of the studies on social media addiction focus on college students and adolescents (Li et al., 2018; Liu and Ma, 2018; Hou et al., 2019; Li et al., 2019; Nie et al., 2019; Chen H.-C. et al., 2020; Li et al., 2020; Wang et al., 2020; Hu et al., 2021). Only a few studies on medical students, nursing students and nurses around the world (Turan et al., 2021; Unal Aslan and Tar, 2021). So far, no research on SMA in Medical care professionals in China has been found. We hope to supplement this data with studies on SMA across the medical care professionals. On the other hand, burnout and self-efficacy have been linked to a number of technology addictions, including Internet addiction, gaming addiction, mobile phone addiction, and so on (Turel and Serenko, 2012). However, few studies have confirmed that these correlations also exist in social media addiction, it is not clear whether these associations are also present in SMA, and the impact intensity of burnout and self-efficacy on social media addiction has not been measured. These critical gaps in the literature suggest that social media addiction research has neglected the medical community or some other professional population, which is detrimental to addressing the problem of social media addiction. Therefore, there are 2 main research objectives of this paper: 1: To investigate the status and influencing factors of SMA addiction in Chinese medical care professionals 2. To analyze whether job burnout and self-efficacy affect SMA levels in Chinese medical care professionals.

## Materials and Methods

### Research Participants

This study has the following screening criteria for participants. The inclusion criteria of participants were as follows: (1) participants must be regular employees who have signed an employment contract with the hospital; (2) participants must be doctors and nurses working on the clinical front line; and (3) participants participated in the survey voluntarily. The excluded criteria of participants were as follows: (1) medical students and nursing students who are practicing in hospitals and (2) managers who do not work in the clinical front line.

### Measures

The measurement items in this study were three validated scales (Social Networking Service Addiction Scale (SNSAS); Maslach’s Burnout Inventory–General Survey (MBI-GS); General Self-efficacy Scale (GSES)) and eight demographic datasets.

#### Social Networking Service Addiction Scale

We measured participants’ levels of SMA using a scale developed by Chinese researchers (Wang, 2019) based on the Bergen Facebook Addiction Scale (Andreassen et al., 2012) and its improved version, the Bergen Social Media Addiction Scale (Andreassen et al., 2017). Their research data came from participants in mainland China and covered the social media commonly used by Chinese people, including QQ, WeChat, and Momo, which meets the needs of this paper. The scale has six dimensions: mood modification, salience, tolerance, withdrawal, conflict, and relapse. The total score of the scale is 5, and the higher the score is, the more serious the participant’s social media addiction is. Each question [e.g., “When I cannot use mobile social networking services (SNS), I crave them more”] is assessed on a five-point Likert scale: 1 strongly agree, 2 basically agree, 3 fair, 4 basically disagree, and 5 strongly disagree. Cronbach’s α coefficient of the scale in this study was 0.893. According to CFA analysis, AVE values of the six dimensions are 0.560–0.762, and CR values are 0.771 ~ 0.906, indicating that the analyzed data have good convergence validity (Table 1).

**Table 1:** CFA test results of Social Networking Service Addiction Scale (SNSAS), Maslach’s Burnout Inventory-General Survey (MBI-GS), and General Self-efficacy Scale (GSES).

Factor	AVE	CR
**SNSAS**		
Mood modification	0.560	0.792
Salience	0.587	0.810
Tolerance	0.567	0.796
Withdrawal	0.762	0.906
Conflict	0.564	0.771
Relapse	0.668	0.857
**MBI-GS**		
Emotional exhaustion	0.697	0.920
Depersonalization	0.709	0.907
Personal accomplishment	0.570	0.888
**GSES**	0.517	0.904

#### Maslach’s Burnout Inventory–General Survey

The MBI-GS is a revised version of the MBI scale. It is a widely used tool for measuring burnout in different work groups in China (Zhong et al., 2009; Guo et al., 2015; Wang et al., 2019; Yuan et al., 2021). This study used the Chinese version of the MBI-GS, which is divided into emotional exhaustion, cynicism, and reduced personal accomplishment (Maslach et al., 1996). Each item (e.g., “Work makes me feel physically and mentally exhausted”) is assessed on a seven-point Likert scale: 0 never; 1 very rarely, several times a year or less; 2 occasionally, once a month or less; 3 often, several times a month; 4 frequently, once a week; 5 very frequently, several times a week; and 6 daily. A higher score corresponded to a stronger burnout. Cronbach’s α coefficient of the MBI-GS in this study was 0.874. According to CFA analysis, AVE values of the three dimensions are 0.697–0.709, and CR values are 0.888–0.920, indicating that the analyzed data have good convergence validity (Table 1).

#### General Self-Efficacy Scale

This study used the GSES (Schwarzer et al., 1997) to measure the self-efficacy level of medical care professionals. The GSES has been widely used in China to collect the self-efficacy values of various social groups (Tan et al., 2015; Zhang and Jin, 2016; Zheng et al., 2016; Zhang et al., 2017). Each item (e.g., “I can always solve problems if I work hard at it”) is assessed on a four-point Likert scale: 1 fully disagree, 2 somewhat agree, 3 mostly agree, and 4 fully agree. A higher score corresponded to a stronger sense of self-efficacy. Cronbach’s α coefficient of the GSES in this study was 0.903. GES is a single dimension scale, corresponding AVE value is 0.517, and CR value is 0.904, indicating that the analysis data has good convergence validity (Table 1).

We also identified eight demographic variables that may have impacted the data through expert consultation: occupation (doctor or nurse), gender, age, marital status, professional title, income satisfaction, sleep quality, and work experience. Among them, income satisfaction and sleep quality were each queried using a single question: “How satisfied are you with your current income?” “How was your sleep quality in the past month?,” respectively, and their answers were assessed on a five-point Likert scale.

### Data Collection

**Figure 2:**
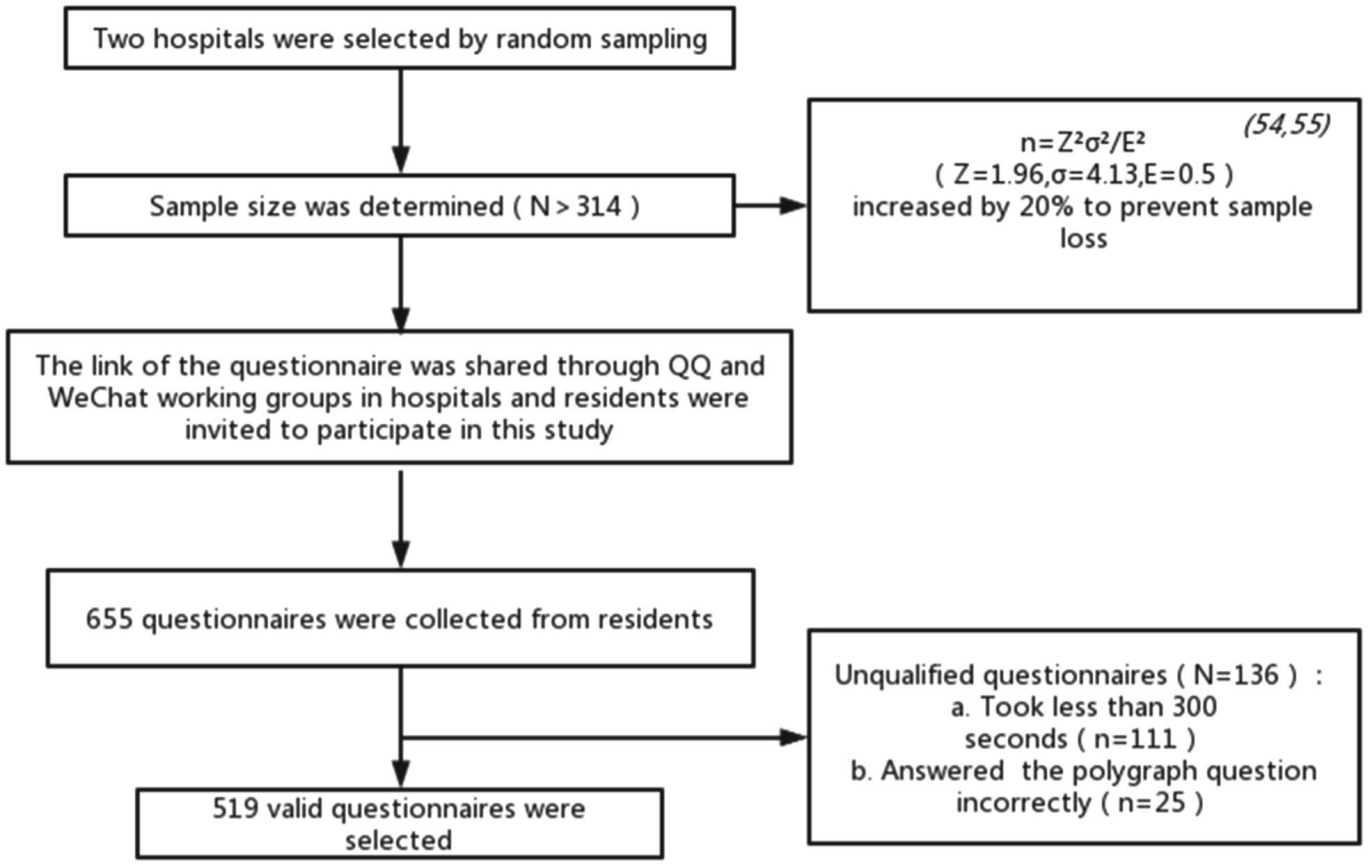
Participant sampling and recruitment process.

We conducted a pre-survey prior to the formal survey, to ensure the accuracy of a formal investigation. We have collected 50 questionnaires in July 2021, and modified the questionnaire based on the results to remove three irrelevant questions: “Do you use mobile phones before going to bed” and “how many hours do you sleep every day?” “And” Are you pursuing a graduate degree?.” We use the sampling function of EXCEL software, using a simple sampling method to randomly select two hospitals in Changsha City, Hunan Province, and survey the formal medical care professionals of the two hospitals. The two hospitals have about 3,600 doctors and nurses. According to the standard deviation of SMA scores in existing surveys in China, which is 4.13 (Hou et al., 2019), the required sample size was calculated (Fang, 2010; Sun and Xu, 2014). An additional 20% was included to prevent sample loss. The results showed that more than 314 samples would meet the study needs. We prepared questionnaires and conducted the survey using “WJX. CN,” a popular platform for Chinese research,^1^ and distributed the questionnaires to hospital medical staff through social media such as QQ and WeChat. The sampling and recruitment process is shown in Figure 2. This would have ensured that every potential participant would receive the questionnaire, but on a voluntary basis, we received 655 questionnaires in total, exceeding the sample size required, and stopped distributing the questionnaire. At last, we received 519 valid questionnaires. “WJX” is widely used in questionnaire research in China. It is efficient and reliable (Peng et al., 2021).

### Quality Control

The survey was completely anonymous and was conducted from August 6 to August 25, 2021. The screening criteria for invalid questionnaires were as follows: (1) completing the questionnaire in less than 300 s (lower than the normal answering time) and (2) answering polygraph questions incorrectly. Healthcare providers who were interested in participating in the study received an online informed consent statement. All procedures were approved by the institutional review board of the Third Xiangya Hospital of Central South University. A total of 655 participants completed the survey, and 136 invalid questionnaires were excluded, leaving 519 valid questionnaires (79.24%).

### Statistical Analysis

SPSS 24.0 was used for data analysis. The *T*-test was used to analyze the differences of SMA levels in occupation, sex and marital status. Analysis of variance was used to examine the relationship between SMA scores and discontinuous variables, such as Professional position. Pearson’s correlation coefficient was used to measure the correlation between continuous variables such as SMA, burnout and self-efficacy, and linear regression analysis was further used to test the effect of independent variables on SMA outcome. Statistical significance was set at *p* < 0.05 in this paper.

## Results

### Sociodemographic Characteristics of the Participants

Table 2 lists all sociodemographic characteristics and the corresponding means and standard deviations of the SMA scores. Among the 519 medical care professionals surveyed, nurses constituted slightly more than half of the population (56.07%), females accounted for 75.92% of the population, the age distribution was 18–50 years, and most participants were married (59.73%). A total of 253 people (48.73%) had junior titles, 181 people had intermediate titles (34.87%), and 85 people had senior titles (16.38%). Nearly half of the respondents (47.21%) believed that their income was medium, and some were dissatisfied with their income (15.22%) or very dissatisfied (8.67%). A total of 51.06% of the respondents thought that their sleep quality was fair, 18.30% reported poor sleep quality, and 8.48% reported very poor sleep quality. Most respondents (58.77%) had more than 5 years of work experience.

**Table 2:** Characteristics and SNSAS scores of the respondents.

Characteristic	*N*	%	SNSAS score	
			Mean	SD
**Occupation**				
Doctor	228	43.93	2.94	0.90
Nurse	291	56.07	2.86	0.81
**Gender**				
Male	125	24.08	2.82	0.90
Female	394	75.92	2.92	0.83
**Age**				
18–25	152	29.29	3.04	0.80
26–30	68	13.10	3.07	0.85
31–40	202	38.92	2.85	0.86
41–50	83	15.99	2.68	0.86
>50	14	2.70	2.52	0.74
**Marital status**				
Single (includes divorced and widowed)	195	37.57	3.03	0.80
Married	310	59.73	2.81	0.87
**Professional position**				
Without title	128	24.66	3.02	0.81
Primary title	125	24.08	3.02	0.91
Middle title	181	34.87	2.81	0.79
Vice senior title	70	13.49	2.66	0.92
Senior title	15	2.89	2.97	0.83
**Income satisfaction**				
Extremely unsatisfied	45	8.67	3.32	0.80
Basically satisfied	79	15.22	2.93	0.78
Neutral	245	47.21	2.86	0.85
Basically unsatisfied	127	24.47	2.79	0.85
Quite satisfied	23	4.43	2.94	0.93
**Sleep quality**				
Very good	17	3.28	2.84	0.62
Good	98	18.88	3.18	0.84
Medium quality	265	51.06	2.81	0.88
Poor	95	18.30	2.94	0.79
Very poor	44	8.48	2.73	0.79
**Working years**				
<1	137	26.40	3.03	0.74
1–3	46	8.86	3.17	0.97
3–5	31	5.97	2.96	0.93
>5	305	58.77	2.79	0.85

### Distribution of the SNSAS, MBI-GS, and GSES Scores of the Participants

The SMA score of the participants was 2.898 ± 0.849 (Table 3), and the scores for tolerance (3.231 ± 1.197) and mood modification (3.210 ± 1.048) were the highest. The current SMA scale can measure only the degree of SMA, and no clear standard is available for SMA (Wang et al., 2019). A total of 357 participants’ (68.79%) scores (Table 4) reached 2.5 points (half of the highest possible score), 113 participants’ (21.77%) scores reached 3.5 points, and 20 participants’ (3.9%) scores reached 4.5 points, indicating that SMA scores of Chinese medical care professionals were relatively high. In addition, the burnout score of the participants was 2.230 ± 0.941, which is a relatively high overall score. The general self-efficacy score of the participants was 2.345 ± 0.524, indicating strong overall self-efficacy.

**Table 3:** Scores on the SNSAS, MBI-GS, and GSES scales.

Scale	Mean	SD	IQR
**SNSAS**	2.898	0.849	1.111
Mood modification	3.210	1.048	1.500
Salience	2.812	1.012	1.500
Tolerance	3.231	1.197	2.000
Withdrawal	2.813	1.129	2.000
Conflict	2.813	1.138	2.000
Relapse	2.368	1.086	1.000
**MBI-GS**	2.230	0.941	1.267
Emotional exhaustion	2.515	1.203	1.200
Depersonalization	1.743	1.304	1.500
Personal accomplishment	2.316	1.398	2.333
**GSES**	2.345	0.524	2.390

SD, standard deviation; IQR, interquartile range.

**Table 4:** Distribution of SNSAS scores (*n* = 519).

Score	*x* ≥ 2.5	*x* ≥ 3.5	*x* ≥ 4.5	Total
*N*	357	113	20	519
%	68.79	21.77	3.9%	100

SD, standard deviation.

### The Relationship Between Demographic Variables and SMA Scores

As can be seen from the data in Table 5, we performed *T*-tests for occupation, gender and marital status. The results show that occupation (*p* > 0.05, Cohen’s *d* = 0.091) and gender (*p* > 0.05, Cohen’s *d* = 0.125) were not associated with SMA levels. However, SMA levels were different in different marital status (*p* < 0.01, Cohen’s *d* = 0.267). Through variance analysis, we found that SMA level was highly correlated with age (*p* < 0.01), professional title (*p* < 0.01), and working years (*p* < 0.01). As shown in Table 4, participants aged 26–30 had the highest SMA score, followed by those aged 18–25. The SMA scores of unmarried participants were significantly higher than those of married participants. The SMA scores of participants without professional titles and those with junior professional titles were higher than those of participants with other professional titles, and the deputy directors had the lowest SMA score. Participants with 1–3 years of work experience had the highest SMA scores, followed by those with less than 1 year of work experience, and those with more than 5 years of work experience had the lowest scores. The Pearson correlation coefficients are given in Table 6. Income satisfaction was negatively correlated with SMA (*p* < 0.01) and the four dimensions of salience (*p* < 0.01), tolerance (*p* < 0.05), withdrawal (*p* < 0.05), and relapse (*p* < 0.01). Sleep quality was negatively correlated with the SMA score (*p* < 0.05). Burnout was negatively correlated with self-efficacy (*p* < 0.01), income satisfaction (*p* < 0.01), and sleep quality (*p* < 0.01). According to the above data analysis results, the two analysis items of occupation and gender do not support H1. But the differences of social media addiction scores in marital status, age, professional position and working years support H1.

**Table 5:** Results of *t*-test and analysis of variance of SNSAS score.

Characteristic		SNSAS total	Mood modification	Salience	Tolerance	Withdrawal	Conflict	Relapse	Cohen’s *d*
**Occupation** (*T*-test)	*t*	1.031	0.431	0.684	0.316	1.027	0.358	2.261	0.091
	*p*	0.303	0.666	0.494	0.752	0.305	0.720	0.024^*^	
**Gander** (*T*-test)	*t*	−1.214	−1.397	−1.627	−1.796	−0.967	0.283	0.850	0.125
	*p*	0.225	0.163	0.104	0.073	0.334	0.777	0.395	
**Marital status** (*T*-test)	*t*	3.036	2.022	4.669	3.606	1.433	1.501	−0.075	0.267
	*p*	0.003^**^	0.044^*^	0.000^**^	0.000^**^	0.152	0.134	0.940	
**Age** (ANOVA)	*F*	4.085	2.194	9.122	6.354	0.811	1.527	0.188	
	*p*	0.003^**^	0.069	0.000^**^	0.000^**^	0.518	0.193	0.945	
**Professional position** (ANOVA)	*F*	3.359	3.184	6.877	3.791	1.064	2.195	0.446	
	*p*	0.010^**^	0.013^*^	0.000^**^	0.005^**^	0.374	0.068	0.776	
**Working years** (ANOVA)	*F*	4.463	2.138	6.795	5.570	2.729	2.779	1.525	
	*p*	0.004^**^	0.094	0.000^**^	0.001^**^	0.043^*^	0.041^*^	0.207	

* *p* < 0.05;

** *p* < 0.01.

ANOVA, analysis of variance.

**Table 6:** Pearson correlation analysis of self-efficacy, income satisfaction, sleep quality, and SNSAS scores.

C	SNSAS	Mood modification	Salience	Tolerance	Withdrawal	Conflict	Relapse	MBI-GS	GSES	Income satisfaction
SNSAS	1									
Mood modification	0.751^**^	1								
Salience	0.858^**^	0.589^**^	1							
Tolerance	0.751^**^	0.460^**^	0.678^**^	1						
Withdrawal	0.843^**^	0.466^**^	0.613^**^	0.584^**^	1					
Conflict	0.706^**^	0.379^**^	0.523^**^	0.464^**^	0.574^**^	1				
Relapse	0.667^**^	0.387^**^	0.469^**^	0.334^**^	0.564^**^	0.506^**^	1			
MBI-GS	0.314^**^	0.241^**^	0.297^**^	0.250^**^	0.214^**^	0.187^**^	0.275^**^	1		
GSES	−0.039	0.010	−0.071	−0.086	−0.020	−0.034	0.012	−0.347^**^	1	
Income satisfaction	−0.123^**^	−0.062	−0.142^**^	−0.101^*^	−0.089^*^	−0.060	−0.121^**^	−0.331^**^	0.105^*^	1
Sleep quality	−0.089^*^	−0.063	−0.085	−0.054	−0.058	−0.079	−0.085	−0.282^**^	0.104^*^	0.235^**^

* *p* < 0.05;

** *p* < 0.01.

### Linear Regression Analysis of Influencing Factors of Social Media Addiction

The Pearson correlation coefficients are given in Table 6. SMA score was significantly positively correlated with burnout (*p* < 0.01), income satisfaction (*p* < 0.01) and sleep quality (*p* < 0.05). We further found that each SMA dimension score was positively correlated with burnout score (*p* < 0.01). The GSES was not correlated with SMA (*p* > 0.05). On the basis of the above data analysis, we performed linear regression analysis for SMA score and burnout, as well as demographic variables (Table 7). As the results showed, burnout significantly positively affected SMA (*p* < 0.01). The linear regression also shows us that each dimension of burnout had a positive impact on all six dimensions of SMA (*p* < 0.01; Table 8), indicating that burnout has a significant positive impact on SMA. The link between SMA score and burnout score proves H2. However, social media addiction score was not related to self-efficacy score, and this result could not support H3.

**Table 7:** Linear regression analysis of the SNSAS (*n* = 519).

	B	SE	Beta	t	p	95% CI	VIF
Constant	2.654	0.234	−	11.326	0.000^**^	2.195–3.113	−
MBI-GS	0.271	0.041	0.300	6.621	0.000^**^	0.191–0.351	1.197
Sleep quality	−0.022	0.041	−0.024	−0.536	0.592	−0.103–0.059	1.146
Income satisfaction	0.003	0.040	0.003	0.075	0.940	−0.076–0.082	1.195
Age	−0.068	0.056	−0.093	−1.211	0.226	−0.179–0.042	3.400
Professional position	−0.041	0.053	−0.053	−0.775	0.439	−0.145–0.063	2.711
Working years	−0.010	0.049	−0.015	−0.196	0.844	−0.105–0.086	3.327

** *p* < 0.01.

Constant: SNSAS score.

**Table 8:** Linear regression results between SMA and burnout dimensions.

Variables	Emotional exhaustion		Cynicism		Reduced personal accomplishment	
	*B*	*p*	*B*	*p*	*B*	*p*
Mood modification	0.203	<0.01	0.158	<0.01	0.087	<0.01
Salience	0.163	<0.01	0.172	<0.01	0.161	<0.01
Tolerance	0.180	<0.01	0.133	<0.01	0.172	<0.01
Withdrawal	0.137	<0.01	0.160	<0.01	0.113	<0.01
Conflict	0.116	<0.01	0.134	<0.01	0.107	<0.01
Relapse	0.167	<0.01	0.218	<0.01	0.130	<0.01

## Discussion

### Relationships Between Sociodemographic Characteristics and SMA Among Medical Care Professionals

This study is one of the earliest studies on SMA among Chinese medical care professionals. The data showed that the SMA score of the participants was 2.898 ± 0.849, which is a relatively high overall score. One study found that nurses in Turkey were also affected by SMA (Hosgor et al., 2021), but there were no studies in the physician population for reference. In addition, many studies in college students found that the incidence of SMA was already higher among medical staff during their school years (Turan et al., 2021; Yilmazel, 2021; Ilter and Ovayolu, 2022). The scores for mood modification (3.210 ± 1.048) and tolerance (3.231 ± 1.197) were the highest, indicating that medical care professionals mainly use social media to avoid work problems and relieve anxiety and depression. SMA among Chinese medical care professionals warrants more attention.

The results of the “occupation” and “gender” data analysis failed to support our first hypothesis. No significant difference in SMA scores was found between doctors and nurses. With little data from physician populations available from other studies, this finding may need to be backed up by more research. Gender did not affect SMA, which is consistent with some studies (Tang and Koh, 2017; Yu and Luo, 2021), although other studies have shown that SMA may have gender differences (Aslan and Yasar, 2020). Since the female sample in this paper is more than the male sample, the influence of medical gender on social media addiction deserves further discussion.

Differences in SMA levels in age, gender, professional position, and working time support H1. There are many demographic differences in SMA levels in medical care professionals. Significant differences in SMA by age, work experience, and professional title were noted. Young medical care professionals between 18 and 30 years old had the highest SMA scores. This age group is more familiar with the use of the internet and smart devices (Wang, 2019). SMA levels are relatively high in the young population (Andreassen, 2015; Lenhart, 2015). Work experience was also correlated with SMA. The SMA level of medical staff with less than 3 years of working experience was significantly higher than that of staff with more working experience. A total of 183 healthcare professionals had less than 3 years of experience in this survey, almost all of whom were 18–30 years old (93.99%). Similarly, the SMA scores of employees with lower professional titles were higher than those of employees with higher professional titles. The SMA level of unmarried medical care professionals was significantly higher than that of married respondents, mainly because their scores for salience, tolerance, and conflict were significantly different. The scores on questions such as “Mobile SNS takes a lot of my time” and “I am obsessed with my mobile SNS” were significantly higher in unmarried medical care professionals (*p* < 0.01), implying that they were more obsessed with social media, that they spent more time on social media, and that social media had more impact on their lives. The unmarried group relies more on social media to communicate with the outside world, but married people may prefer face-to-face communication with family members. Previous studies have reported a relationship between addictive behaviors and family support, and people lacking family support have a higher risk of internet addiction (Isik and Ergun, 2018). Family support can reduce the risk of internet addiction (Gunuc and Dogan, 2013). In the treatment of internet addiction, family support plays an important role (Senormanci et al., 2014; Liu et al., 2015), which can reduce the impact of internet addiction (Isik and Ergun, 2018). SMA was negatively correlated with income satisfaction. The number of medical care professionals who thought that their income was fair, unsatisfactory, or very unsatisfactory reached 369 (71.1%). SMA differs with income (Aydin et al., 2021). Low-income groups are more prone to internet addiction (Muller et al., 2014). Studies have also found that adolescents with low family income are more susceptible to internet addiction (Wu et al., 2016) and smartphone addiction (Bhanderi et al., 2021). The sleep quality of medical care professionals was negatively correlated with SMA. A total of 404 (77.84%) people indicated that their sleep quality was not good (fair, poor, or very poor). On the one hand, technology addiction can reduce sleep efficiency (Sumen and Evgin, 2021), and sleep problems are a risk factor for burnout (Soderstrom et al., 2012). On the other hand, burnout can lead to higher cognitive and emotional arousal, which are core elements of the vicious cycle of chronic insomnia (Rothe and Specht, 2021). Burnout will further aggravate SMA. However, this paper only selected some of the items most likely to affect the level of SMA in medical care professionals for collection, as there is a lack of more studies in the same population for reference, there may be more influencing factors to be discovered.

### Effect of Burnout Among Medical Care Professionals on SMA

In line with our second hypotheses, the second main finding of this study is that burnout was positively correlated with SMA, which is consistent with some previous studies; that is, stress is positively correlated with technology addiction (Chen et al., 2014; Jun and Choi, 2015; Younes et al., 2016; Radeef and Faisal, 2018; Gong et al., 2021). The regression results showed that each dimension of burnout had a positive impact on the six dimensions of SMA, and this positive impact was still significant under the influence of multiple demographic variables. A study in China suggests that limiting mobile phone use among young nurses may reduce burnout (Ma et al., 2021).

The data in this study showed that the emotional exhaustion score of the burnout scale was the highest (2.515 ± 1.203). Most healthcare providers (53.56%) felt physically and mentally exhausted because of their work several times a month or more, exhausted at the end of the day (53.95%), tired of facing work in the morning (44.12%), stressed out from working all day (43.35%), and drained due to work (21.58%). Through a review of the literature, we found that a heavy workload is the main source of stress for medical care professionals (Ye et al., 2019), which includes writing too many prescriptions (Yang et al., 2017) and working long hours (even more than 60 h a week; Guo et al., 2016; Wen et al., 2016). Furthermore, the COVID-19 pandemic has imposed additional pressure and has had an overwhelming psychological impact on medical care professionals (Orru et al., 2021), especially physicians caring for critically ill patients (Azoulay et al., 2020) and first-line nurses (Zare et al., 2021).

Correspondingly, the score for the mood modification dimension of SMA among medical care professionals was very high. A total of 41.23% of participants reported using SNS as a way to escape problems and relieve anxiety, and 48.75% patients used SNS to relieve depression. Previous studies have also shown that technology addiction provides an escape from the pressures of life (Chen et al., 2014; Jun and Choi, 2015; Younes et al., 2016; Radeef and Faisal, 2018; Gong et al., 2021). The long hours and high stress of their jobs afford them only fragmented time for entertainment, and using mobile phones to browse social media is undoubtedly the most convenient and efficient.

Based on these findings, we speculate that the high level of social media dependence among healthcare professionals may be related to their work stress and fatigue. Moreover, SMA may further aggravate burnout by affecting factors such as sleep quality (Kawyannejad et al., 2019) and interpersonal relationships (Bevan et al., 2014). There is a bad cycle between SMA and burnout.

### Effect of the GSE of Medical Care Professionals on SMA

Our third hypothesis is that self-efficacy level was negatively correlated with SMA level in Chinese medical care. Two studies from Korea showed that self-efficacy negatively affected SMA levels of Korean students (Seo et al., 2015; Im, 2017). Some studies have also shown that self-efficacy has the potential to negatively affect technology addiction (Iskender and Akin, 2010; Jeong and Kim, 2011; Chiu, 2014; Lee et al., 2018; Chen H. -C. et al., 2020; Berte et al., 2021). Therefore, the GSE of medical care professionals is highly likely to be negatively correlated with SMA. However, the data in this study showed no correlation between the self-efficacy of medical care professionals and SMA, The results of data analysis do not support our H3. First, we questioned whether demographic factors, such as age, interfered with the results. However, after restricting the sample to the 18–30-year-old age group, the Pearson correlation coefficient was 0.013 (*p* > 0.05), and the results were unchanged. The effects of all demographic variables were further excluded, and the results were still consistent, i.e., no correlation was observed between GSE and each dimension of SMA.

This is an unexpected and interesting result. Due to the lack of studies on self-efficacy and technology addiction in medical care professionals, we have reached the tentative conclusion that the level of self-efficacy in the medical care population does not have an impact on SMA. The impact of GSE on other technological addictions in medical care professionals (such as gaming addiction) and the situation in other countries or regions need to be clarified by further research.

## Theoretical Implications

In recent years, most of the studies on social media addiction focus on college students and adolescents Li et al., 2018, 2019, 2020; Liu and Ma, 2018; Nie et al., 2019; Chen I. H. et al., 2020; Wang et al., 2020; Hu et al., 2021). There are only a few studies on medical students, nursing students (Unal Aslan and Tar, 2021) and nurses (Turan et al., 2021) around the world. The first theoretical advance of this study is to extend the study of SMA to the entire medical care professionals community. The level of social media addiction can be influenced by many factors. Since researchers usually over focus on the common mental health problems at present, such as depression and anxiety (Demirci, 2019). But few studies have combined job burnout and self-efficacy with SMA, although in other studies burnout and self-efficacy have been shown to be associated with technology addiction (Chen et al., 2014; Seo et al., 2015; Younes et al., 2016; Im, 2017), therefore, the second theoretical advantage of this study is that combining burnout theory, self-efficacy theory with SMA, and the correlation between burnout and SMA was confirmed. This study reminds other researchers, perhaps many influencing factors of SMA remain unexplored.

## Strengths, Limitations, and Future Research

This study is the earliest study on SMA among Chinese medical care professionals. It is also one of the first SMA studies on medical care professionals worldwide. We found higher SMA scores in the Chinese healthcare professionals and identified demographic factors that contribute to this phenomenon. Our study is the first to identify the impact of burnout on SMA in healthcare workers. Moreover, we found that self-efficacy had no effect on SMA in the healthcare population, which is quite different from previous studies. This study provides a reference for further studies on SMA in medical care professionals and other professional populations and provides additional data for further research on the relationship between SMA and burnout and self-efficacy. It also provides reference for the intervention research of SMA.

No unified diagnostic criteria are available for SMA; therefore, this study cannot clearly classify SMA scores but can only describe the degree of SMA. In this study, the proportion of males was 24.08%, which was due to the strong predominance of females among nurses. Future surveys that expand the sample size of males are needed to further clarify the impact of gender on SMA among medical care staff. In addition, we selected only two hospitals in one city for the survey, which does not reflect the overall reality of SMA among medical care professionals in China. Future studies need to expand the scope and include larger samples.

## Conclusion

This is the first study on SMA among medical care professionals in China. We hope that our study will promote further in-depth analyses of SMA prevention and intervention in medical care populations in the future. First, our study found that the SMA scores of Chinese medical care professionals were relatively high, and the SMA scores were unrelated to occupation and gender. Medical care professionals who are young, unmarried, or dissatisfied with their income, have a low professional title, have less work experience, and have poor sleep quality are at a high risk for SMA. Second, we found a strong association between burnout and SMA, and the influence was still significant when controlling for multiple demographic variables. Therefore, to reduce the SMA level of the medical care population, we should first start with reducing burnout, enabling medical care professionals to achieve sufficient sleep, increasing medical staff income, and providing more opportunities for promotion. It is important to note that self-efficacy shows a negative influence on SMA in some populations, but this effect does not seem to exist in the healthcare population, which deserves further investigation.

## Data Availability Statement

The raw data supporting the conclusions of this article will be made available by the authors, without undue reservation.

## Ethics Statement

The studies involving human participants were reviewed and approved by the Institutional review board (IRB) of the Third Xiangya Hospital, Central South University. The patients/participants provided their written informed consent to participate in this study.

## Author Contributions

AL participated in research design and data analysis. WK participated in data collecting, data analysis, and paper writing. HH and YL participated in data collecting. WX participated in research design, data analysis, and paper writing. All authors contributed to the article and approved the submitted version.

## Funding

This research was supported by the Natural science foundation of Changsha city: Research on tertiary hospitals “Internet + medical” health service quality evaluation and user adoption (kq2014270).

## Conflict of Interest

The authors declare that the research was conducted in the absence of any commercial or financial relationships that could be construed as a potential conflict of interest.

## Publisher’s Note

All claims expressed in this article are solely those of the authors and do not necessarily represent those of their affiliated organizations, or those of the publisher, the editors and the reviewers. Any product that may be evaluated in this article, or claim that may be made by its manufacturer, is not guaranteed or endorsed by the publisher.

## Footnotes

^1^ www.wjx.com
